# EFA-RadNet: Efficient Feature Aggregation with Balanced Attention for Raw Radar Multi-Task Learning

**DOI:** 10.3390/s26072050

**Published:** 2026-03-25

**Authors:** Chengliang Zhong, Xiuping Li, Jingjing Li, Juan Liu, Xiyan Sun

**Affiliations:** 1 Guangxi Key Laboratory of Precision Navigation Technology and Application, Guilin University of Electronic Technology, Guilin 541004, China; chengliabgzhong@mails.guet.edu.cn (C.Z.); lxp863@gmail.com (X.L.); lijingjing@guet.edu.cn (J.L.); liujuan@mails.guet.edu.cn (J.L.); 2 Information and Communication School, Guilin University of Electronic Technology, Guilin 541004, China; 3 National & Local Joint Engineering Research Center of Satellite Navigation Positioning and Location Service, Guilin 541004, China; 4 GUET-Nanning E-Tech Research Institute Co., Ltd., Nanning 530031, China

**Keywords:** millimeter-wave radar, object detection, deep learning, feature aggregation, attention mechanism, autonomous driving

## Abstract

Original high-definition radar data contains rich environmental information, including distance, Doppler velocity, and azimuth. However, extracting robust features from such sparse and noisy frequency-domain data remains a challenge. To address this issue, this paper proposes an improved multi-task network, the Efficient Feature Aggregation with Balanced Attention Radar Network (EFA-RadNet). This network introduces the VoVNetV2 architecture into the field of raw radar perception and effectively preserves feature diversity across different receptive fields through a One-Shot Aggregation (OSA) module, avoiding signal aliasing. In addition, we propose an attention mechanism module, Balanced effective Squeeze–Excitation (B-eSE), which is better suited for sparse radar processing and effectively addresses the problem of weak target loss in the radar spectrum. Experiments on the RADIal dataset show that our EFA-RadNet achieves excellent target detection performance while also attaining optimal accuracy in free space segmentation.

## 1. Introduction

The safe deployment of autonomous driving technology heavily relies on the robustness of the environmental perception system. Although perception schemes primarily based on cameras and Light Detection and Ranging (LiDAR) have made significant progress in recent years [1,2,3], their performance often degrades substantially under adverse weather or extreme lighting conditions. In contrast, millimeter-wave radar, leveraging its longer wavelength characteristics, can penetrate rain and fog, providing reliable all-weather perception capabilities [4]. In particular, with the iteration of hardware technology, the next-generation High-Definition (HD) imaging radar not only provides range and velocity information but also outputs high-resolution elevation and azimuth angular information, generating dense point clouds that gradually narrow the performance gap with LiDAR [5,6].

To fully exploit the rich information from HD radar, a large number of deep learning-based perception methods have emerged in academia. Among them, FFT-RadNet [7], proposed by Rebut et al., is a milestone work. It constructs an end-to-end fully convolutional network that learns multi-task features directly from the complex Range–Doppler (RD) spectrum, avoiding expensive angle Fast Fourier Transform (FFT) computations and information loss. Although FFT-RadNet validates the effectiveness of spectrum-based learning, its feature extractor still employs Residual Network (ResNet) [8], a classic architecture from the computer vision domain. While ResNet excels in processing dense optical images, its core inductive bias does not fully align with the physical characteristics of sparse radar spectrums when applied to sparse and noisy radar data. Specifically, ResNet relies on element-wise summation in residual connections. In radar RD spectrums with extremely low Signal-to-Noise Ratio (SNR), directly summing feature maps containing weak target signals with those dominated by clutter easily leads to feature dilution, causing target signals to be overwhelmed by noise. Furthermore, although attention mechanisms such as Squeeze-and-Excitation Network (SENet) [9] and Convolutional Block Attention Module (CBAM) [10] are widely used for feature recalibration, they typically include dimensionality reduction operations to reduce parameter count. As pointed out by Lee et al. [11], such reduction destroys feature integrity. For sparse radar data, critical Doppler features may be distributed across only a few channels, and dimensionality reduction often leads to irreversible information loss.

Addressing the aforementioned challenges, this paper proposes a novel network architecture optimized for raw radar perception, named Efficient Feature Aggregation with Balanced Attention Radar Network (EFA-RadNet). Inspired by the success of CenterMask [11] in efficient instance segmentation tasks, we adapt and transfer the VoVNetV2 backbone to the radar domain. Unlike the dense connections in DenseNet [12], VoVNetV2 utilizes the One-Shot Aggregation (OSA) [13] module to aggregate multi-scale features via concatenation rather than summation. This mechanism allows the network to preserve original signals from different receptive fields across distinct channels, effectively preventing noise from interfering with weak signals. Furthermore, we propose the B-eSE module. By adopting a non-reduction channel strategy and incorporating dual-stream pooling with layer normalization, this module effectively resolves the numerical scale imbalance problem between sparse weak targets and high-intensity background noise in the radar spectrum.

The main contributions of this paper are as follows:

- Architectural Innovation: A perception network named EFA-RadNet, optimized for raw HD radar, is proposed. Through the introduction of the OSA architecture, the feature dilution problem inherent in ResNet when processing spectral data is effectively addressed;
- Module Innovation: An attention mechanism, B-eSE, tailored for sparse radar signals is developed. Experimental results demonstrate that this mechanism plays a crucial role in improving the recall rate of weak targets;
- Performance Breakthrough: Extensive comparative experiments on the RADIal dataset indicate that the proposed method achieves superior levels of performance in both object detection and free-space segmentation tasks, with significant improvements particularly in Average Recall (AR) and segmentation precision (mIoU).

## 2. Related Work

In recent years, with the growing demand for all-weather environmental perception capabilities in autonomous driving, deep learning algorithms based on millimeter-wave radar have become a research hotspot in both academia and industry. Early research primarily borrowed processing pipelines from LiDAR, first converting radar signals into sparse point clouds via Constant False Alarm Rate (CFAR) detection, and then processing them using point cloud networks such as PointNet [14], PointPillars [3], or Graph Neural Networks [15]. However, this approach suffers from a severe information bottleneck: the threshold truncation in CFAR causes a significant amount of weak echoes containing environmental semantics to be discarded as noise [4]. To address this issue, recent research trends have shifted towards directly processing raw data. For instance, Major et al. [16] utilize raw data for static-dynamic separation; RadarNet [17] explores voxelized radar feature representations; and Radar Object Detection Network (RODNet) [18] proposes cross-modal supervised learning using Range–Doppler maps. FFT-RadNet [7] and its subsequent variants have validated the feasibility of end-to-end learning on compressed RD spectrums. Building on this foundation, recent research is dedicated to extending this paradigm to higher-dimensional perception tasks. For example, RadOcc [19] innovatively expands radar perception from object detection to 3D occupancy prediction, utilizing raw data to achieve dense modeling of dynamic and static environments. Simultaneously, addressing the alignment challenge in multi-modal fusion, Radar-Camera Multi-Level Fusion (RCM-Fusion) [20] proposes a radar-camera multi-level fusion framework, significantly enhancing feature robustness through instance-level contrastive learning. Furthermore, RadarDistill [21] addresses the semantic insufficiency of radar features by introducing a cross-modal knowledge distillation framework, which leverages large vision models as teacher networks to guide the learning and enhancement of radar features, thereby significantly elevating the detection upper bound of single-modality radar.

As a core component for enhancing the representational capability of convolutional neural networks, attention mechanisms have evolved into various variants within the computer vision domain. Early SENet [9] introduced channel attention via the Squeeze-and-Excitation operation, while the CBAM [10] further integrated spatial dimension attention. However, these classic modules were primarily designed to process highly redundant natural images, and thus universally include channel dimensionality reduction operations to control model parameters. Recent studies indicate that such dimensionality reduction designs may disrupt direct channel-to-channel mapping relationships when processing low-redundancy features. Addressing this issue, CenterMask [11] proposed the eSE module, which achieves lossless modeling of full-channel dependencies by removing the dimensionality reduction bottleneck in fully connected layers. Although eSE preserves channel information by eliminating the reduction bottleneck, its reliance on Global Average Pooling tends to smooth feature distributions. In the context of sparse radar data, this easily leads to localized high-energy peaks representing targets being `diluted’ by background clutter in the radar spectrum. Consequently, existing mechanisms still struggle to balance channel fidelity with the acute capture of weak peak features when processing highly sparse and noisy radar signals.

## 3. EFA-RadNet Architecture

**Figure 1:**
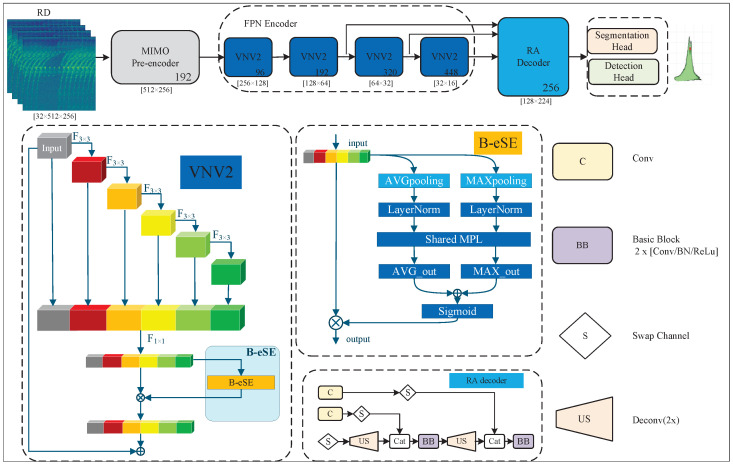
EFA-RadNet overall network structure.

As shown in Figure 1, the network architecture of our proposed Efficient Feature Aggregation with Balanced Attention Radar Network (EFA-RadNet) follows the overall framework of FFTRadNet [7] and is primarily composed of four modules: a MIMO pre-encoder, a Feature Pyramid Network (FPN) integrating the VoVNetV2 backbone with the B-eSE module, a Range-Angle decoder, and two task-specific heads for object detection and free-space segmentation, respectively.

### 3.1. MIMO Pre-Encoder

This paper directly uses the complex Range–Doppler (RD) spectrum as input. To address the interleaving issue of MIMO radar signals along the Doppler dimension, we adopt the MIMO pre-encoder designed in FFT-RadNet [7]. This module comprises an atrous convolution layer tailored for specific Doppler phase shifts, followed by a feature compression convolution layer. It reorganizes and maps the raw complex tensor of dimensions $\left(B_{R},B_{D},N_{\mathrm{Rx}}\right)$ into feature maps suitable for 2D CNN processing, thereby providing a compact input representation for the subsequent feature extraction network while preserving phase information.

### 3.2. FPN Encoder

#### 3.2.1. OSA

The core building block of the VoVNetV2 backbone is the OSA module. The OSA module consists of a series of consecutive convolutional layers and performs a one-time feature aggregation at the end of the module. Formally, let $X_{\mathrm{in}}\in R^{C\times H\times W}$ denote the input tensor of the module. The module contains N consecutive 3×3 convolutional layers, denoted as $L_{1},L_{2},\ldots ,L_{N}$, and aggregates the outputs of all intermediate layers in the final step. Its mathematical expression is defined as follows:

First, generate the intermediate feature hierarchy:

(1) $$\begin{array}{cc} X_{1} & =L_{1}\left(X_{\mathrm{in}}\right) \end{array}$$

(2) $$\begin{array}{cc} X_{i} & =L_{i}\left(X_{i-1}\right),\;i=2,\ldots ,N \end{array}$$

Subsequently, a one-shot aggregation operation is performed. All intermediate feature maps are concatenated with the input feature map along the channel dimension:

(3) $$X_{\mathrm{agg}}=\left[X_{\mathrm{in}},X_{1},X_{2},\ldots ,X_{N}\right]$$

where [·] denotes the concatenation operation along the channel axis. At this stage, the channel count of $X_{\mathrm{agg}}$ increases significantly, encapsulating features from varying depths.

Finally, a 1×1 convolutional layer is applied to fuse the aggregated features and adjust the channel dimensionality:

(4) $$X_{\mathrm{OSA}}={\mathrm{Conv}}_{1\times 1}\left(X_{\mathrm{agg}}\right)$$

Since the radar RD spectrum is characterized by significant sparsity and typically low Signal-to-Noise Ratio (SNR), target signals often manifest as weak energy peaks while the background is dominated by strong clutter. In contrast to ResNet’s processing of sparse radar spectrums, the OSA module employs an aggregation strategy based on concatenation rather than summation. This approach effectively addresses the issue where radar signals are low in SNR and easily masked by clutter, preventing weak target signals from being diluted or aliased by background noise, thereby ensuring signal integrity. Furthermore, OSA synchronously aggregates features from different levels, endowing the output tensor $X_{\mathrm{OSA}}$ with both small receptive fields from shallow layers and large receptive fields from deep layers. This synchronous encoding of multi-scale spatial-frequency information significantly enhances the model’s perceptual robustness across targets of varying sizes.

#### 3.2.2. Balanced Effective Squeeze-Excitation (B-eSE)

A critical challenge in processing raw radar spectrums lies in suppressing background clutter while preserving sparse target features. Although a dual-pooling structure has proven effective in the CBAM [10], its internal dimensionality reduction bottleneck is destructive to radar signals. On the other hand, while the eSE module proposed in CenterMask [11] preserves channel integrity by removing this reduction operation, it relies solely on Global Average Pooling (GAP) to aggregate spatial information. Radar targets in RD spectrum typically manifest as localized high-energy peaks, whereas the background appears as widely distributed low-energy noise. GAP tends to smooth the overall feature distribution, which can easily cause weak target peaks to be ’diluted’ by the background noise floor. In contrast, Global Max Pooling (GMP) is more sensitive to salient feature responses and can effectively lock onto high-frequency target signals.

To address this, we propose an improved module distinct from the traditional CBAM and eSE, named Balanced effective Squeeze–Excitation (B-eSE) (as shown in Figure 1). This module integrates a dual-path pooling strategy with a layer normalization mechanism while strictly maintaining non-reduction in channel dimensions. Specifically, we employ GAP and GMP in parallel to extract both the global background distribution and local salient features. Addressing the extreme numerical scale imbalance between Max (high-energy peaks) and Avg (low-energy noise floor) inherent in radar data, directly adding unnormalized features, as is done in the standard CBAM, would result in the gradients being dominated by the massive Max values. To resolve this radar-specific dilemma, we introduce Layer Normalization (LN) [22] after pooling, a critical component absent in traditional attention mechanisms. LN acts as an adaptive gain controller here, forcing the two feature paths into a unified distribution and effectively preventing the gradients of the subsequent Shared Fully Connected Layer (Shared FC) from being entirely dominated by the large-value Max branch. This mechanism successfully activates the Avg branch’s perception of background levels, prompting the network to learn feature selection based on relative Signal-to-Noise Ratio (SNR) rather than absolute intensity. Furthermore, the design of abandoning dimensionality reduction ensures that critical channels carrying unique phase or micro-Doppler information in sparse radar signals are not lost, thereby achieving more robust feature recalibration.

(5) $$\begin{array}{cc} F_{\mathrm{avg}} & =\mathrm{LN}\left(\mathrm{GAP}\left(X_{\mathrm{OSA}}\right)\right)\in R^{C\times 1\times 1} \end{array}$$

(6) $$\begin{array}{cc} F_{\max} & =\mathrm{LN}\left(\mathrm{GMP}\left(X_{\mathrm{OSA}}\right)\right)\in R^{C\times 1\times 1} \end{array}$$

To model the non-linear dependencies between channels, we feed the features from both branches into a Shared FC. In our implementation, this Shared FC is constructed using a weight-shared 1×1 convolutional layer.

(7) $$\begin{array}{cc} M_{\mathrm{avg}} & ={\mathrm{Conv}}_{1\times 1}\left(F_{\mathrm{avg}};W_{\mathrm{shared}}\right) \end{array}$$

(8) $$\begin{array}{cc} M_{\max} & ={\mathrm{Conv}}_{1\times 1}\left(F_{\max};W_{\mathrm{shared}}\right) \end{array}$$

where $W_{\mathrm{shared}}\in R^{C\times C}$ denotes the shared learnable weight matrix. Sharing weights not only reduces the number of parameters but, more importantly, forces the network to learn a channel correlation pattern that is generalizable across different statistical features (i.e., mean and extreme values). Finally, the outputs of the two branches are fused via element-wise addition, and a Sigmoid activation function σ is applied to generate the final channel attention map, which is then used to re-weight the original input, where ⊗ indicates element-wise multiplication:

(9) $$\begin{array}{c} A=\sigma \left(M_{\mathrm{avg}}+M_{\max}\right) \end{array}$$

(10) $$\begin{array}{c} Y_{B-\mathrm{eSE}}=X_{\mathrm{OSA}}⊗A \end{array}$$

#### 3.2.3. Residual Connection

While the OSA module preserves feature diversity via concatenation, the complex non-linear distribution of radar data necessitates a deeper network for high-level semantic extraction. To address optimization challenges caused by gradient attenuation in deep networks, VoVNetV2 integrates a Macro-Residual Connection strategy. Unlike traditional layer-wise micro-residuals, this connection spans the entire OSA module and the subsequent B-eSE module. We fuse the recalibrated features $Y_{B-\mathrm{eSE}}$ with the original input $X_{\mathrm{in}}$ via identity mapping:

(11) $$Y_{\mathrm{out}}=Y_{B-\mathrm{eSE}}+X_{\mathrm{in}}$$

This mechanism effectively mitigates network degradation, ensuring smooth gradient flow and enabling EFA-RadNet to robustly parse complex radar spectrums through deeper non-linear transformations.

### 3.3. RA Decoder

To transform the multi-scale features from the FPN into a range–azimuth (RA) representation suitable for downstream tasks, we adopt the decoder structure proposed in [7]. Since azimuth information is encoded in the channel dimension within the backbone, while spatial dimensions correspond to range and Doppler, the decoder’s core task is axis permutation and spatial upsampling. Specifically, a 1×1 convolution first adjusts the channel count, followed by an axis swap operation that exchanges the Doppler and azimuth axes, converting the feature map from $\left(B_{R},B_{D},B_{A}\right)$ to $\left(B_{R},B_{A},B_{D}\right)$. To restore the range dimension downsampled during encoding, deconvolution layers are employed to upsample only the range axis, fusing shallow FPN features via skip connections to preserve spatial details. Finally, the network outputs a high-resolution RA latent.

### 3.4. Multi-Task Head

The Segmentation Head and Detection Head proposed in [7] are adopted in this work to perform the object detection and semantic segmentation tasks.

## 4. Experimental Results and Analysis

### 4.1. Datasets

To validate the performance of the proposed model, this work employs the automotive millimeter-wave radar benchmark dataset RADIal [7] for experimentation. The core acquisition device is a radar system operating under the DDMA-MIMO scheme, equipped with 12 transmitting and 16 receiving units, capable of synthesizing up to 192 virtual apertures. Simultaneously, the dataset utilizes a high-precision 16-beam LiDAR and an RGB camera for cross-modal joint calibration, providing rich annotation information that covers range, azimuth, and 2D image bounding boxes. The entire dataset comprises 8252 synchronized samples, divided into a training set (6231 frames), a validation set (986 frames), and a test set (1035 frames).

### 4.2. Simulation Setup

Regarding evaluation metrics, we adhere to the standards established in [7], selecting Average Precision (AP), Average Recall (AR), mean Intersection over Union (mIoU), and model parameters as the primary criteria. Additionally, range error (ΔR) and angle error (ΔA) are introduced to quantitatively assess target localization accuracy. The final model selection is based on the comprehensive performance of the F1-score and mIoU.

The hyperparameters for the training process are configured as follows: the Adam optimizer is utilized for parameter updates with an initial learning rate set to $10^{-4}$, employing a step-wise decay strategy where the rate is multiplied by 0.9 every 10 epochs. The entire training cycle spans 100 epochs, with the dataset partitioned into training, validation, and test sets in a 7:1.5:1.5 ratio.

### 4.3. Result

#### 4.3.1. Comparison with State-of-the-Art Models

Table 1 presents the detection performance comparison between EFA-RadNet and other state-of-the-art models. Experimental results demonstrate that EFA-RadNet achieves comprehensive superiority over the baseline FFTRadNet, with the most significant breakthrough in AR, which surges from 82.20% to 90.42% (an increase of 8.22%). Notably, while substantially boosting recall, the model also attains an AP of 97.22% (+0.42%) and an F1-score of 93.70% (+4.8%). This indicates that the model effectively captures more targets without introducing additional false positives, achieving a synergistic optimization of detection precision and breadth. Furthermore, it maintains exceptionally high standards in localization errors (ΔR and ΔA).

Table 2 presents the performance evaluation results of different models on the free-space segmentation task. As shown in the Table 2, EFA-RadNet achieves a mIoU of 82.19%, realizing a significant performance improvement of 8.19% compared to the baseline model FFTRadNet. Furthermore, compared to state-of-the-art models such as T-FFTRadNet, Cross Modal DNN, and ADCNet, EFA-RadNet also demonstrates optimal segmentation performance, fully demonstrating its superiority in perceiving drivable areas within complex scenarios.

#### 4.3.2. Qualitative Results

**Figure 2:**
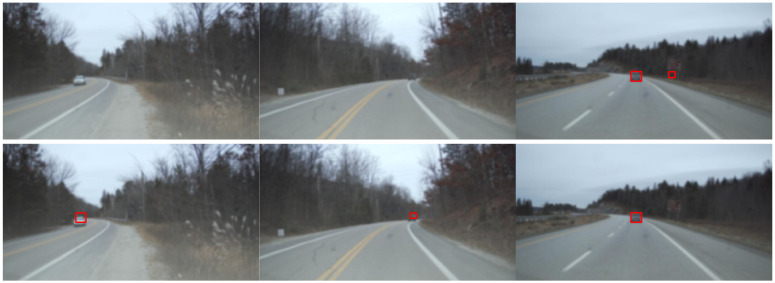
Qualitative comparison between our model and FFTRadNet. Top row: results from FFTRadNet. Bottom row: results from our model.

As shown in Figure 2, the left and middle columns demonstrate weak target recall scenarios where FFTRadNet fails to detect distant vehicles (indicated by missing red boxes). In contrast, the proposed model EFA-RadNet successfully recalls these targets by acutely capturing localized weak energy peaks. This is achieved because the OSA module preserves multi-scale original signal details through feature concatenation. Subsequently, the Max-Pooling branch within the B-eSE module acts as a highly sensitive probe, acutely locking onto these localized weak energy peaks within a uniformly normalized feature space, thereby ensuring perceptual continuity in low Signal-to-Noise Ratio scenarios. The right column showcases an interference suppression scenario where FFTRadNet misclassifies a strongly reflecting metal sign as a vehicle. Conversely, the proposed model EFA-RadNet effectively distinguishes between static clutter and real targets. This demonstrates the synergistic effect of LN and the Shared FC within the B-eSE module, rather than merely searching for the highest energy point, it incorporates the global average background to learn to discriminate the authenticity of targets based on relative SNR and micro-Doppler features. Consequently, it exhibits exceptional anti-interference robustness in highly noisy environments.

#### 4.3.3. Complexity Analysis

Table 3 presents the theoretical floating-point operations (FLOPs) and number of parameters for different models, calculated by averaging over the test set. Compared to the baseline FFT-RadNet [7], our EFA-RadNet introduces the VoVNetV2 backbone and the B-eSE module, resulting in slightly higher parameter count and computational complexity. However, this marginal computational investment yields a massive leap in overall perception performance (e.g., an 8.22% increase in Average Recall), demonstrating that EFA-RadNet possesses exceptionally high representation efficiency. Furthermore, compared to other state-of-the-art models, our method achieves superior detection and segmentation performance while maintaining lower parameter counts and computational complexity.

Additionally, to evaluate practical deployment feasibility, we conducted an assessment of actual real-time inference speeds for the baseline FFTRadNet and our EFA-RadNet under identical hardware conditions (a single NVIDIA RTX 3090 GPU, Batch Size = 20). The results show that the baseline achieves an average inference latency of 15.76 ms (63.44 FPS), whereas EFA-RadNet records an average latency of 16.45 ms (60.79 FPS). This indicates that our proposed modules introduce a negligible latency overhead of only approximately 0.7 ms, which is trivial in practical applications and can meet the strict real-time requirements of autonomous driving. In summary, EFA-RadNet achieves an optimal balance among precision, recall, and computational efficiency, making it a highly competitive solution for raw radar perception.

### 4.4. Ablation Studies

To validate the effectiveness of the proposed method in sparse radar data processing tasks, we conducted an ablation study on the VNVB module design with different attention mechanisms, as well as a multi-component ablation experiment on the B-eSE architecture. The structure of the VNVB module, as shown in Figure 1, consists of VoVNetV2 and B-eSE. The ablation results for the VNVB module and the B-eSE architecture are presented in Table 4 and Table 5, respectively.

As shown in Table 4, compared to the standard eSE module, the proposed B-eSE further improves AR by 0.90% (reaching 90.42%) and significantly boosts mIoU by 1.57%. These performance gains validate the rationality of the B-eSE design.

As shown in the ablation experimental results of the B-eSE module in Table 5, although the standalone Max branch exhibits high sensitivity to high-energy peaks, it is susceptible to strong clutter interference in low Signal-to-Noise Ratio environments, resulting in deviation from real targets and missed detection of weak and small targets. The standalone Avg branch imposes a smoothing effect on features, which also masks the effective signals of weak targets. Meanwhile, the numerical magnitude imbalance between the Max branch (high-energy peaks) and Avg branch (low-energy background noise) in sparse radar data tends to cause the gradient updates of the Shared Fully Connected (FC) Layer to be dominated by the Max branch. However, the LayerNorm layer normalizes the features from the Max and Avg branches into a unified distribution space, effectively addressing the numerical magnitude imbalance between the two branches and preventing the Shared FC from favoring high-energy signals indiscriminately. On this basis, the model learns to adaptively balance the weight allocation between background suppression (Avg) and target enhancement (Max), thereby enabling robust extraction of weak target signals that are easily corrupted by strong noise and diluted by background in radar spectrograms and achieving more robust feature recalibration. Furthermore, the ablation study in Table 5 demonstrates that the enhancements in AR, AP, and mIoU further verify the rationality of the proposed B-eSE module.

## 5. Conclusions

This paper proposes a novel network architecture named EFA-RadNet for raw high-definition radar perception tasks. Through an in-depth analysis of the limitations of ResNet in processing sparse radar spectrums, we introduce and adapt the VoVNetV2 backbone and propose the B-eSE module. Our research finds that the feature concatenation mechanism of the OSA module effectively addresses the feature dilution problem caused by the layer-wise summation in ResNet, preserving multi-scale original signal features. Meanwhile, the B-eSE module successfully resolves the issue of weak signals being diluted by background clutter, acting as an efficient spectral filter. Experimental results on the RADIal dataset demonstrate that, with a controllable number of parameters, this method significantly improves the detection recall (AR) and segmentation precision (mIoU), validating the effectiveness of EFA-RadNet.

## Figures and Tables

**Table 1:** Detection performance comparison of our method with state-of-the-art approaches.

Methods	AP	AR	F1	ΔR	ΔA
FFTRadNet [7]	96.80%	82.20%	88.90%	0.12 m	0.10°
T-FFTRadNet [23]	89.60%	89.50%	89.50%	0.15 m	0.12°
Cross Modal DNN [24]	96.90%	83.50%	89.70%	-	-
ADCNet [25]	95.00%	89.00%	91.90%	0.13 m	0.10°
EFA-RadNet	**97.22%**	**90.42%**	**93.70%**	0.12 m	0.10°

Note: The bold values indicate the best performance in the column.

**Table 2:** Performance of free-space segmentation task.

Methods	mIoU
FFTRadNet [7]	74.00%
T-FFTRadNet [23]	80.20%
Cross Modal DNN [24]	80.40%
ADCNet [25]	78.95%
EFA-RadNet	**82.19%**

Note: The bold values indicate the best performance in the column.

**Table 3:** Complexity analysis result.

Methods	Parameters	Complexity
FFTRadNet [7]	3.79 M	288 G
T-FFTRadNet [23]	9.64 M	194 G
Cross Modal DNN [24]	7.7 M	358 G
EFA-RadNet	6.52 M	320 G

**Table 4:** Ablation study on different attention mechanisms in the VNVB module.

VOVNetV2	eSE	B-eSE	AP	AR	F1	mIoU
✓			96.91%	88.46%	92.49%	79.64%
✓	✓		97.06%	89.52%	93.14%	80.62%
✓		✓	**97.22%**	**90.42%**	**93.70%**	**82.19%**

Note: The bold values indicate the best performance in the column.

**Table 5:** Ablation study of different modules in the B-eSE architecture.

AVGPooling	MAXPooling	Shared FC	LN	AP	AR	F1	mIoU
✓				97.06%	89.52%	93.14%	80.62%
	✓			96.82%	89.21%	92.86%	81.16%
✓	✓	✓		97.08%	89.31%	93.03%	81.61%
✓	✓	✓	✓	**97.22%**	**90.42%**	**93.70%**	**82.19%**

Note: The bold values indicate the best performance in the column.

## Data Availability

The raw data supporting the conclusions of this article will be made available by the authors on request.
